# Facile Route to Achieve a Hierarchical CuO/Nickel-Cobalt-Sulfide Electrode for Energy Storage

**DOI:** 10.3390/mi14112095

**Published:** 2023-11-13

**Authors:** Sa Lv, Zhifei Cheng, Yaodan Chi, Huan Wang, Xuefeng Chu, Yang Zhao, Boqi Wu, Runsheng Wang, Zhiwen Zhang, Chao Wang, Jia Yang, Xiaotian Yang

**Affiliations:** Key Laboratory for Comprehensive Energy Saving of Cold Regions Architecture of Ministry of Education, Jilin Jianzhu University, Changchun 130118, China

**Keywords:** copper oxide, composite, electrodeposition, energy storage

## Abstract

Herein, a novel self-supporting CuO/nickel-cobalt-sulfide (NCS) electrode was designed in a two-step electrodeposition technique followed by a calcination process. Three-dimensional copper foam (CF) was exploited as the current collector and spontaneous source for the in situ preparation of the CuO nanostructures, which ensured sufficient deposition space for the subsequent NCS layer, thus forming abundant electrochemical active sites. Such a hierarchical structure is conducive to providing a smooth path for promoting electronic transmission. Therefore, the optimized CuO/NCS electrode exhibits outstanding energy storage capability with extremely superior specific capacitance (*C*s) of 7.08 F cm^−2^ at 4 mA cm^−2^ and coulombic efficiency of up to 94.83%, as well as excellent cycling stability with capacitance retention of 83.33% after 5000 cycles. The results presented in this work extend our horizons to fabricate novel hierarchical structured electrodes applied to energy storage devices.

## 1. Introduction

In recent years, metal hydroxides/oxides, as pseudocapacitive electrode materials, have attracted extensive attention due to their ultra-high theoretical specific capacitance (*C*_S_). In particular, Ni(OH)_2_ and Co(OH)_2_ have become the preferred pseudocapacitive electrode materials due to their high redox activity, low cost and environmental friendliness [1,2,3]. Ni(OH)_2_ and Co(OH)_2_ with various morphologies and structures have been regulated and constructed on nickel foam substrate through various synthetic paths [4,5]. In addition, exploration has gradually expanded to other optional electrode materials and electrode substrates. Among them, copper foam (CF) is favored by researchers as a current collector and spontaneous source for the direct growth of Cu(OH)_2_. CF can reduce the contact resistance and make the growth environment stable owing to the evenly distributed and dense copper source; therefore, the repeatability of the experiment can be significantly improved, and the generated Cu(OH)_2_ can have desirable electrical conductivity. For example, He et al. designed a facile surface oxidation method to prepare Cu(OH)_2_ nanorods on CF, which exhibited high *C*_S_ of 1.75 F cm^−2^ at 2 mA cm^−2^ [6]. Wong et al. prepared Cu(OH)_2_ nanorods using the anodic oxidation method on copper foil [7]. In addition, Cu(OH)_2_ can be calcined at a high temperature to obtain CuO, which can also be used as electrode material. Apart from maintaining the original rod-shaped structure of Cu(OH)_2_, CuO forms a porous structure due to the evaporation of water, which is more conducive to electron transfer [8]. The above examples confirm that the Cu(OH)_2_ electrode obtained by in situ oxidation technology has a uniform and stable morphology, strong adhesive force with CF substrate and good experimental repeatability. 

In fact, in order to further improve the overall electrochemical properties of the electrodes, many strategies have been proposed to improve the electrochemical activity of Cu(OH)_2_/CuO, including structure tailoring, compositional regulation and heteroatom doping [9,10,11]. Among them, component composition is the most intuitive evaluation and regulation method that can increase the active site of the electrode material, thereby increasing the *C*s of the electrode. For example, Deng et al. synthesized CuO@cobalt-nickel double hydroxides (CoNi LDH) by combining wet chemical oxidation and electrodeposition [12]. The purpose of designing a core–shell structure composite is to generate and expose richer electrochemical active sites to the electrolyte and facilitate charge transport. By implementing a similar strategy, Liu prepared a CuO/NiFe LDH composite electrode. These CuO-based composite electrodes exhibit superior electrochemical performance and cycling stability [13].

Herein, a CuO/nickel-cobalt-sulfide composite electrode was successfully designed and constructed on CF substrate through an electrodeposition technique followed by a calcination process (denoted as CuO/NCS). CuO, as an active electrode component, gave full play to its skeleton support role to provide greater growth space for the deposition of NCS, thereby generating more abundant electrochemical active sites. The selection and composite strategy of this component can effectively enhance the electrochemical behavior of the composite electrode, which provides guidance for the design of a novel hierarchical functional material for utilization in energy storage. 

## 2. Experimental

### 2.1. Materials

CF (130 PPI, 1.5 mm thick) was purchased from the Power Source Battery Sales Department in Taiyuan, China. The treatment of CF includes three steps: (1) cutting the size into 1 × 1.5 cm; (2) cleaning by using hydrochloric acid, ethanol and water thoroughly; (3) vacuum drying. The reagents involved in the preparation process include NaOH, C_12_H_25_SO_3_Na, NiCl_2_·6H_2_O, Co(NO_3_)_2_·6H_2_O and CH_4_N_2_S; they were all analytical grade and purchased from Beijing Chemical Works Co., Ltd. (Beijing, China).

### 2.2. Stepwise Electrodeposition of CuO/NCS Electrode 

The electrochemical deposition process is performed via an electrochemical workstation (Chenhua, CHI 760E). In the first step, a two-electrode setup comprising CF (working electrode) and Pt foil (counter electrode) was employed. The electrolyte was a mixed solution of 2 M NaOH and 0.025 M C_12_H_25_SO_3_Na. After galvanostatic deposition of 0.05 A was performed for 600 s, blue-green Cu(OH)_2_ was produced, followed by calcination at 200 °C for 2 h to generate black CuO. 

The deposition of NCS in the next step is achieved by a three-electrode setup comprising the previously generated CuO (working electrode), saturated calomel electrode (SCE, reference electrode) and Pt foil (counter electrode). The electrolyte was a mixed solution of 0.05 M NiCl_2_·6H_2_O, 0.05 M Co(NO_3_)_2_·6H_2_O and 0.5 M CH_4_N_2_S. After the potentiostatic deposition of −1.1 V was executed for 15 min, a black hierarchical CuO/NCS electrode was obtained. In addition, other deposition times, including 1, 5, 10 and 20 min, were also carried out for comparison, and the corresponding samples were labeled as S-1, S-5, S-10, S-15 and S-20. 

### 2.3. Characterization 

The prepared CuO/NCS electrode was characterized by XRD (Cu Kα radiation with λ = 1.5406 Å), SEM (JSM-7610F 15.0 kV) and XPS (ESCALAB 250Xi Al Kα as X-ray source). Energy storage performance was assessed using cyclic voltammetry (CV), galvanostatic charge–discharge (GCD) and cycling performance with the same electrochemical workstation as above. The three-electrode setup comprising CuO/NCS (working), Ag/AgCl (reference) and Pt foil (counter electrode) was measured in a 2 M NaOH solution. 

## 3. Results

**Figure 1:**
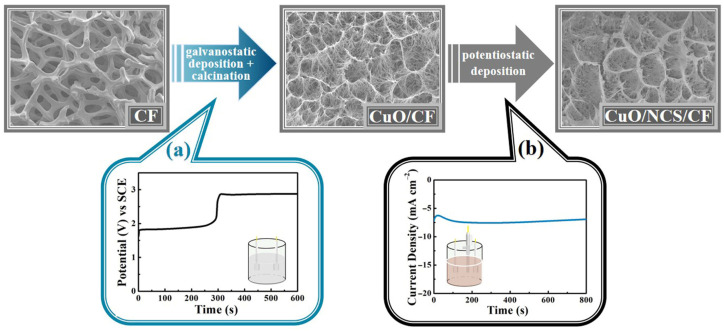
The design process of the CuO/NCS electrode: (**a**) galvanostatic deposition/calcination; (**b**) potentiostatic deposition.

As illustrated in Figure 1, the first step of galvanostatic deposition allowed the in situ oxidation of the 3D CF surface to generate Cu(OH)_2_, which was further calcined at a high temperature to obtain black CuO. The second step was to achieve a dense NCS layer tightly wrapped on the CuO surface via potentiostatic deposition, ultimately generating the hierarchical CuO/NCS electrode.

**Figure 2:**
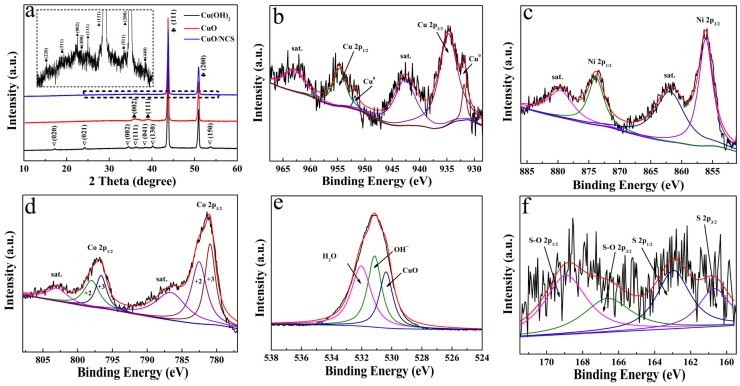
(**a**) XRD patterns of the Cu(OH)_2_, CuO and CuO/NCS electrodes; XPS spectra of the CuO/NCS electrode: (**b**) Cu 2p, (**c**) Ni 2p, (**d**) Co 2p, (**e**) O 1s and (**f**) S 2p.

XRD and XPS measurements were applied to analyze the composition and valence states of the CuO/NCS electrode. As depicted in Figure 2a, the XRD patterns of the three samples all contain two extremely strong diffraction peaks, which can be attributed to the CF substrate [JCPDS 01-1241]. In addition, seven diffraction peaks in the black curve marked with a triangle belong to Cu(OH)_2_ [JCPDS 13-0420]. After calcination, it transforms into two characteristic peaks (marked with spades in the red curve) of CuO [JCPDS 05-0661]. To ensure a clearer diffraction peak of the CuO/NCS electrode, local amplification was adopted as the insertion pattern and the diffraction peak was marked with a square, appearing at 26.9°, 31.6°, 38.3°, 50.3° and 55.1°, which could be ascribed to the (220), (311), (400), (511) and (440) planes of the NCS layer (JCPDS 43-1477) [14,15], thus confirming the successful recombination of CuO and NCS. Figure 2b–f show the XPS spectra of the CuO/NCS electrode. As for the Cu 2p spectrum, two peaks at 954.50 and 934.60 eV originate from Cu 2p_1/2_ and Cu 2p_3/2_, respectively, whereas two peaks at 952.45 and 932.20 eV can be attributed to Cu^0^ from the CF substrate. The other two peaks at 962.93 and 943.00 eV are satellite peaks in Figure 2b [12,16]. For the Ni 2p spectrum, the peaks at 873.80 and 856.10 eV are the characteristics of Ni 2p_1/2_ and Ni 2p_3/2_; the energy separation of 17.7 eV indicates the presence of Ni^2+^ [17,18]. The other two peaks at 879.70 and 861.70 eV are their corresponding satellite peaks (Figure 2c). Both Co 2p_1/2_ and Co 2p_3/2_ signals can be fitted into two peaks, and the peaks at 798.00 and 782.60 eV represent Co^2+^ while the peaks at 796.50 and 781.00 eV represent Co^3+^ [19,20]. The other two peaks at 803.05 and 786.85 eV are their corresponding satellite peaks (Figure 2d). In the O 1s spectrum in Figure 2e, the three decomposed peaks at 532.80, 531.20 and 530.70 eV are derived from H_2_O, OH^−^ and CuO, respectively [13]. The S 2p spectrum in Figure 2f can be divided into two pairs of peaks. One pair at binding energies of 162.80 and 160.70 eV can be ascribed to S 2p_1/2_ and S 2p_3/2_, respectively, and the other pair at 168.90 and 166.60 eV can be ascribed to S-O 2p_1/2_ and S-O 2p_3/2_, respectively [21,22]. The emergence of the S-O bond originates from the combination of S and OH^-^ generated via thiourea hydrolysis [23].

**Figure 3:**
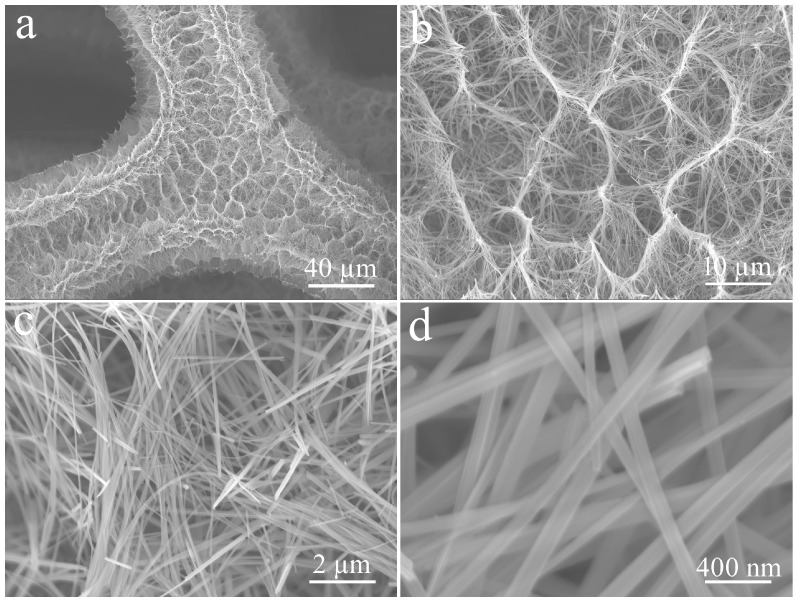
Low (**a**,**b**) and high (**c**,**d**) magnifification FE-SEM images of Cu(OH)_2_ at different magnifications.

The microstructure and surface morphology of electrodes at different preparation stages were evaluated via SEM. Figure 3 shows Cu(OH)_2_ grown on CF obtained in the first stage. From the low magnification images, the dense Cu(OH)_2_ uniformly covers the entire CF substrate in the form of numerous scattered bird nests (Figure 3a,b). It appears that these nests are composed of a large number of Cu(OH)_2_ nanorods with smooth surfaces, which are arranged in an orderly manner, with a diameter of ca. 100 nm (Figure 3c,d).

**Figure 4:**
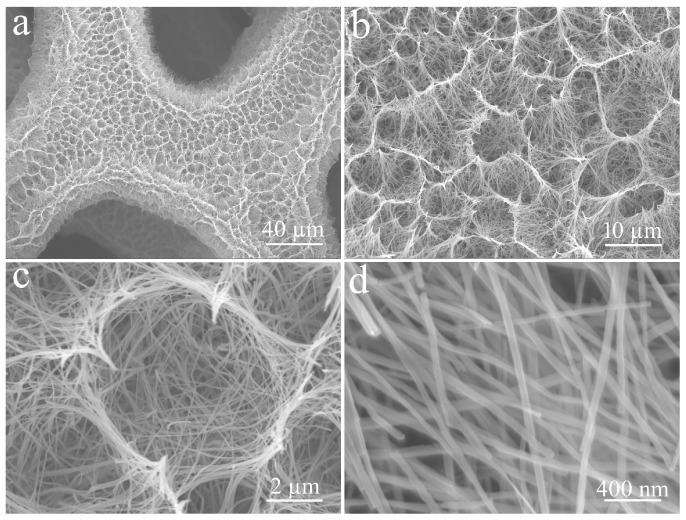
Low (**a**,**b**) and high (**c**,**d**) magnifification FE-SEM images of CuO at different magnifications.

CuO obtained from high-temperature calcination of Cu(OH)_2_ still maintains its original dense nest-like structure, as shown in Figure 4. However, the magnified observation reveals that these nanorods become significantly bent due to high-temperature dehydration.

**Figure 5:**
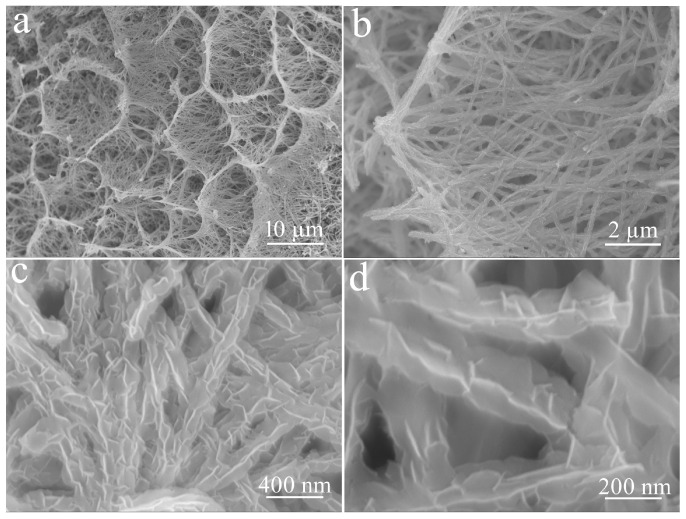
Low (**a**,**b**) and high (**c**,**d**) magnifification FE-SEM images of CuO/NCS at different magnifications.

The exhibited morphology of CuO/NCS is shown in Figure 5. From the low magnification images in Figure 5a, one can find that the surrounding area of these nest-like structures became denser after the second step of the potentiostatic deposition. Enlarging the sidewall of nest-like structure leads to the discovery that the surface of these CuO nanorods became rough (Figure 5b). In fact, they are wrapped by a thick layer of NCS nanosheets with a thick of ca. 9 nm, and they are slightly curved and hinged to each other, similar to the raised edges on the surface of CuO nanorods (Figure 5c,d).

**Figure 6:**
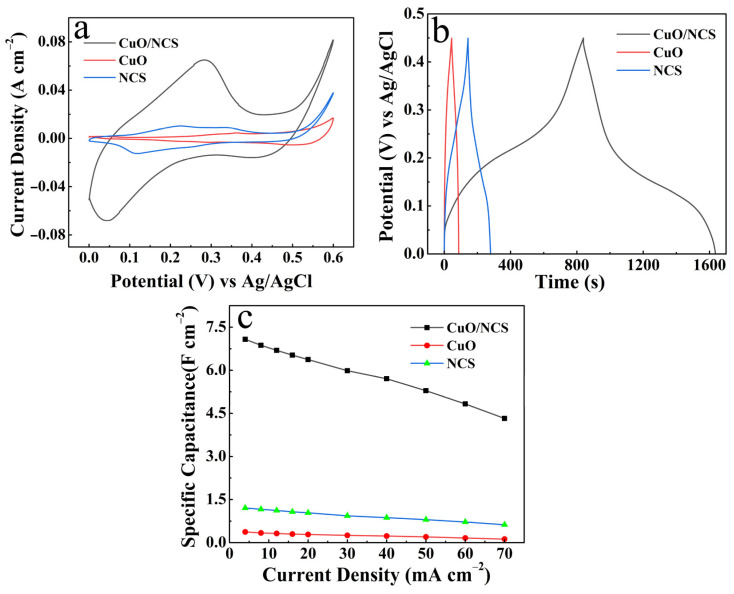
Comparison of electrochemical properties of the CuO/NCS, CuO and NCS electrodes: (**a**) CV curves at 5 mV s^−1^; (**b**) GCD curves at 4 mA cm^−2^; (**c**) *C*s at different current densities.

Figure 6 presents the electrochemical characteristics of the CuO/NCS, CuO and NCS electrodes. Among them, Figure 6a records the CV curves of the three electrodes (5 mV s^−1^), and the CuO/NCS electrode exhibited the strongest electrochemical response with the largest integrated area of the CV curve corresponding to the strongest electrochemical energy storage capability. The inference is also confirmed by the GCD curves in Figure 6b. At the same discharge current density of 4 mA cm^−2^, the CuO/NCS electrode exhibited the longest discharge time. Specifically, the *C*s values calculated for the three electrodes at different discharge current densities are shown in Figure 6c, and the *C*s of the CuO/NCS electrode is much higher than that of the single component electrode.

**Figure 7:**
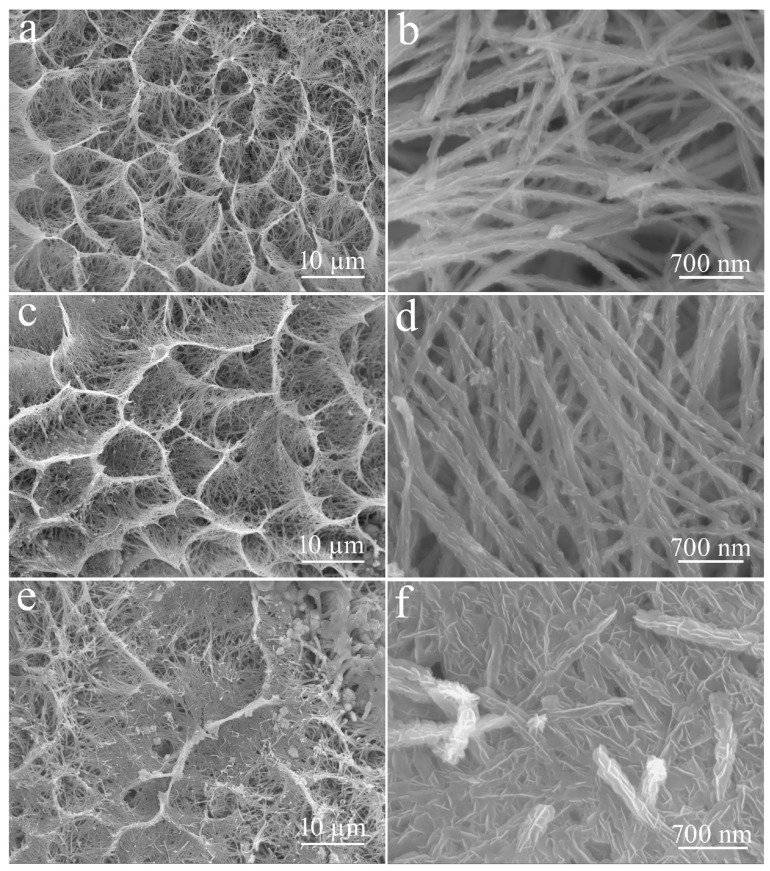
FE-SEM images of NCS electrodeposited on CuO for different execution times: (**a**,**b**) 5 min, S-5; (**c**,**d**) 10 min, S-10; (**e**,**f**) 20 min, S-20.

**Figure 8:**
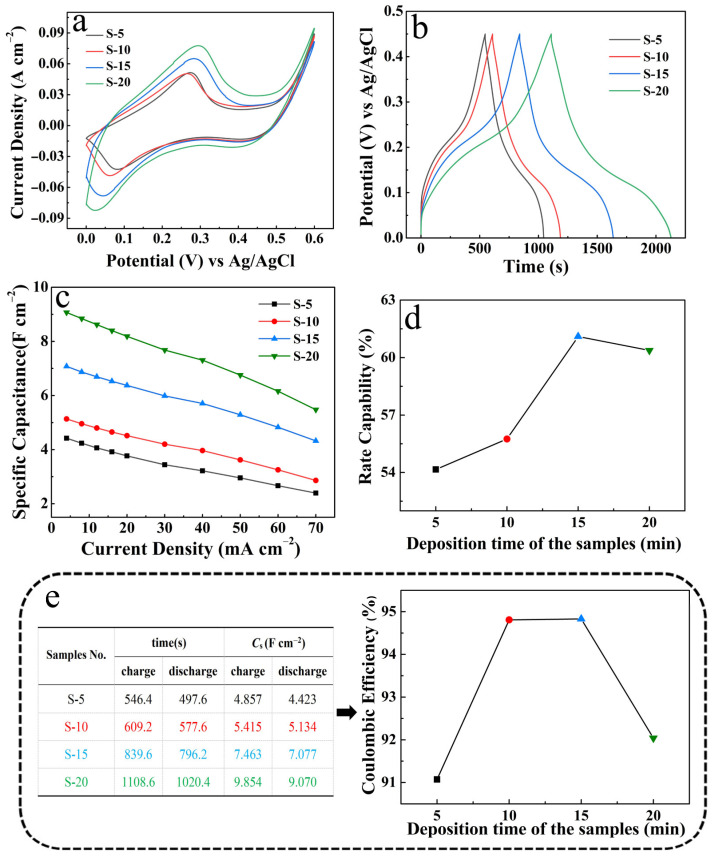
Comparison of electrochemical properties of S-5, S-10, S-15 and S-20: (**a**) CV curves at 5 mV s^−1^; (**b**) GCD curves at 4 mA cm^−2^; (**c**) *C*s at different current densities; (**d**) rate capability; (**e**) coulombic efficiency.

**Figure 9:**
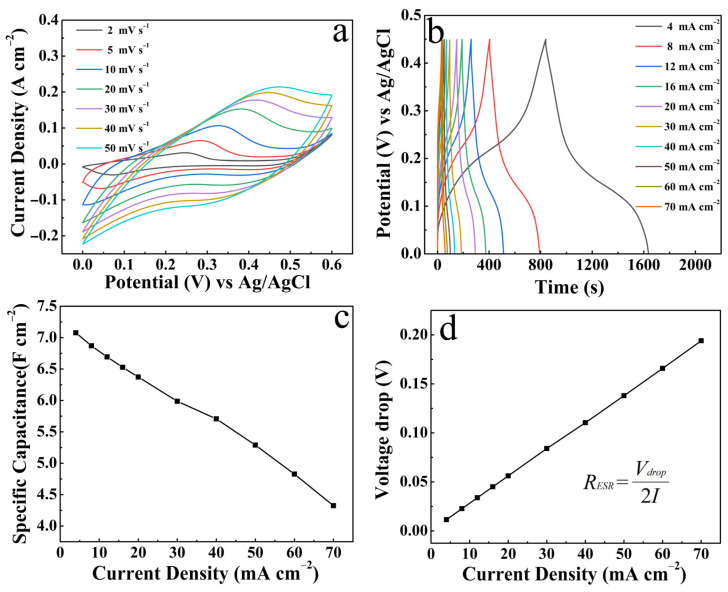
Electrochemical properties of S-15: (**a**) CV curves; (**b**) GCD curves; (**c**) *C*s; (**d**) voltage drop at different current densities.

**Figure 10:**
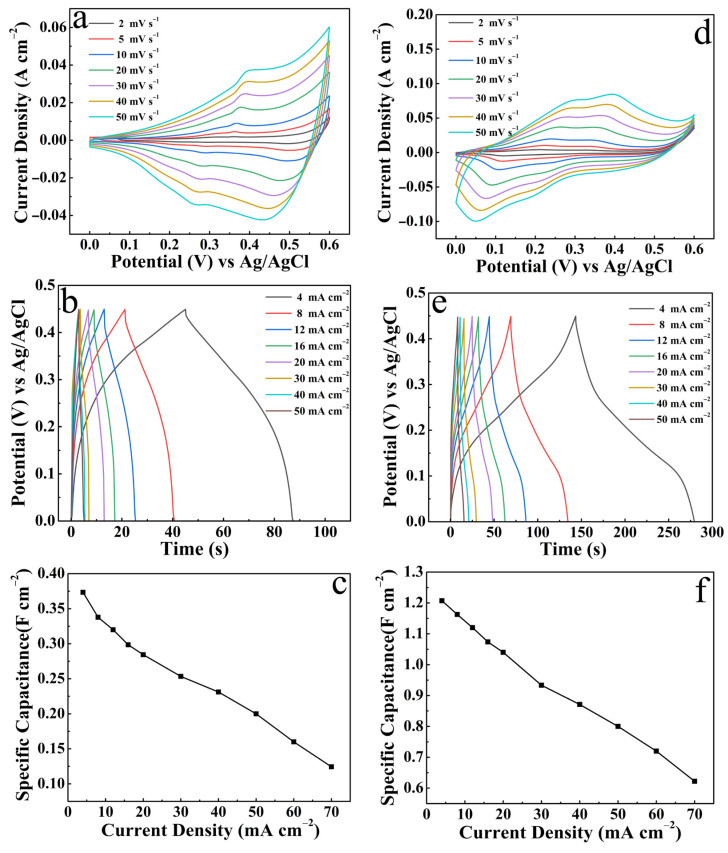
Electrochemical properties of single CuO and NCS: (**a**,**d**) CV curves; (**b**,**e**) GCD curves; (**c**,**f**) *C*s at different current densities.

To further optimize the structure and performance of the CuO/NCS electrode, deposition parameters were regulated. Because of the weak energy storage characteristics of CuO, the amount of NCS deposited on the CuO surface was tuned by adjusting the deposition time, and the morphology of the corresponding products is shown in Figure 7. In fact, when NCS was deposited for just 1 min (S-1), the CuO surface became significantly rougher, as shown in Figure S1. When the NCS deposition time was set at 5 min (S-5), the sidewalls of some nested structures gradually became dense because the CuO surface has been wrapped by a layer of NCS (Figure 7a,b). Further, as depicted in Figure 7c,d (S-10), the thickness of the wrapping layer gradually increased with deposition time. When the NCS deposition time reached 15 min (S-15), it became clear that a layer of curved NCS nanosheets was coated on the CuO surface, as shown in Figure 5. Continuing to extend the NCS deposition time to 20 min (S-20), the excessive deposited of NCS completely collapsed and the CuO nest-like structure was destroyed (Figure 7e,f). 

Figure 8 systematically compares the electrochemical performance of the four electrodes mentioned above. From the CV curves in Figure 8a, at the same scan rate of 5 mV s^−1^, the longer the NCS deposition time, the larger the area of the integration curve, and hence the larger the *C*s. The GCD curves also confirm this trend (Figure 8b). Figure 8c lists the *C*s of the four electrodes at different current densities. It is evident that the energy storage capacities of the four electrodes obey the following sequence: S-20 > S-15 > S-10 > S-5. Meanwhile, the rate capability of the four electrodes can also be calculated and derived from the four sets of *C*s comparison curves. As shown in Figure 8d, the rate capability gradually increased upon increasing the deposition time at the first deposition time of 15 min, and it reached a maximum of 61.10% (S-15). After that, the rate capability decreased when continuing to extend the deposition time. The coulombic efficiency data in Figure 8e also reflect this same trend, with S-15 giving the highest coulombic efficiency of 94.83%. The decrease in performance is attributed to excessive accumulation and collapse of the NCS layer in the S-20 electrode, which is consistent with the morphology characterization in Figure 7e,f. Based on the electrode morphology and performance analysis above, the NCS deposition time was determined to be 15 min. 

Figure 9a shows the CV curves of the CuO/NCS electrode (S-15), which reflects a symmetrical redox peak, and the peak response gradually increased with the increase in the scan rate, but the *C*s gradually decreased. This phenomenon stems from the fact that the slower the scan rate, the more electrochemical active sites are present and this allows sufficient redox reaction time with OH^-^ in the NaOH electrolyte [24]. Figure 9b displays the GCD curves of S-15 at different discharge current densities. The rule is that the shorter the discharge current density, the longer the discharge time. Therefore, according to the formula [25] SI, when the current density increased from 4 to 70 mA cm^−2^, the *C*s value decreased from 7.08 to 4.32 F cm^−2^. The specific correspondence between the current density and the *C*s is shown in Figure 9c. In addition, the average *R*_ESR_ of the CuO/NCS electrode is deduced to be 1.38 Ω cm^−2^ according to the formula in Figure 9d. The outstanding energy storage capability of the CuO/NCS electrode comes from the electrochemical reactions as follows [20,26]. It can be seen that CuO, the sulfides of cobalt and nickel all act as active electrode components participating in pseudocapacitive reactions with the electrolyte (2 M NaOH).

(1) $$\begin{array}{l} 2\mathrm{CuO}+2H_{2}O+2e^{-}↔Cu_{2}O+2OH^{-}+H_{2}O\\ ↔2\mathrm{CuOH}+2OH^{-}↔\;2\mathrm{Cu}{\left(\mathrm{OH}\right)}_{2}+2e^{-} \end{array}$$

(2) $$\mathrm{NiS}+OH^{-}↔\mathrm{NiSOH}+e^{-}$$

(3) $$\mathrm{CoS}+OH^{-}↔\mathrm{CoSOH}+e^{-}$$

(4) $$\mathrm{CoSOH}+OH^{-}↔\mathrm{CoSO}+H_{2}O+e^{-}$$

More importantly, in the structural design process of CuO/NCS electrodes, CuO nanorods fully play a supporting role and provide sufficient deposition space for NCS, thus forming abundant electrochemical active sites. It is obvious from Figure S2, that NCS with irregular nanosheet structure are scattered and stacked on the CF surface in the presence of CuO support.

In fact, we also compared the electrochemical performance of single CuO and NCS electrodes, including CV curves, GCD curves and corresponding *C*s values, as shown in Figure 10. All confirm that the energy storage capability of any single component is far weaker than the CuO/NCS electrode. The conclusion has been elaborated on in Figure 6c.

**Figure 11:**
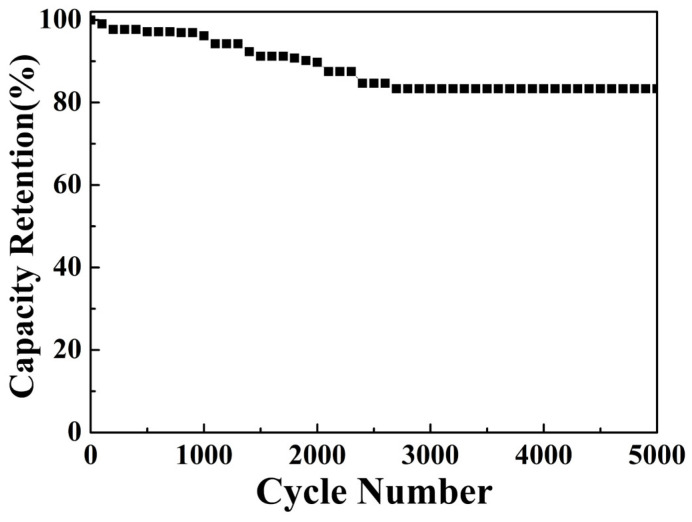
Cyclic stability of the CuO/NCS electrode.

The cyclic stability of the CuO/NCS electrode was further evaluated by adopting 5000 cycles of GCD testing. As seen in Figure 11, the *C*s decreased by nearly 3.85% in the first 1000 cycles. At the 2700th cycle, the *C*s reached 83.33% of the initial value and remained constant. Moreover, there was no significant collapse in the CuO/NCS electrode morphology after testing (Figure S3).

The improved electrochemical performance of the CuO/NCS electrode can be attributed to the follow issues: (1) As a collector, the 3D CF has the advantages of stability and compactness. Meanwhile, this in situ oxidation growth reduces contact resistance [25]. (2) The nested structure formed by CuO nanorods is uniform and dense; although its energy storage capability is weak, it can provide solid support for further deposition of the NCS layer and improve the cycling stability of the electrode [13]. (3) The NCS layer facilitates the regulation of its deposition amount by changing the deposition time [14,27]. In addition, the uniform and dense nanosheet structure ensures the formation of unobstructed electron transport channels [28].

## 4. Conclusions

In conclusion, a rational two-step electrodeposition strategy followed by a calcination process is proposed to construct a hierarchical CuO/NCS electrode. Benefiting from the supporting effect of CuO and abundant electrochemical active sites formed by NCS deposition, the synergistic advantages promote the electrochemical performance of the CuO/NCS electrode, which provides excellent *C*s of 7.08 F cm^−2^ at 4 mA cm^−2^ and coulombic efficiency of up to 94.83%. The cycle stability is maintained at 83.33% of the initial *C*s value within 5000 cycles. Furthermore, this design concept provides an effective reference for the selection of composite electrode components and the construction path of a hierarchical structure, which is widely applied in the field of energy storage and catalysis.

## Data Availability

Data are contained within the article and Supplementary Materials.

## Supplementary Materials

The following supporting information can be downloaded at: https://www.mdpi.com/article/10.3390/mi14112095/s1, Figure S1: Low (a) and high (b) magnifification FE-SEM images of NCS electrodeposited on CuO for 1 min (S-1); Formula for calculating the *C*s; Figure S2: Low (a) and high (b,c) magnifification FE-SEM images of individual NCS deposited on CF at different magnifications.; Figure S3: Low (a) and high (b) magnifification FE-SEM images of CuO/NCS at different magnifications after electrochemical performance testing.
