# Supplementary material for: Facile Route to Achieve a Hierarchical CuO/Nickel-Cobalt-Sulfide Electrode for Energy Storage
Source: Micromachines (Basel). 2023 Nov 13;14(11):2095. doi: 10.3390/mi14112095 (PMC10672988; doi:10.3390/mi14112095)
Supplement: Supplementary file 1 [file micromachines-14-02095-s001.zip › micromachines-2665769-supplementary.pdf]

Supplementary Materials

Facile Route to Achieve a Hierarchical CuO/Nickel-Cobalt-Sulfide Electrode for Energy Storage

Sa Lv *, Zhifei Cheng, Yaodan Chi, Huan Wang, Xuefeng Chu, Yang zhao, Boqi Wu, Runsheng Wang, Zhiwen Zhang, Chao Wang, Jia Yang and Xiaotian Yang *

Key Laboratory for Comprehensive Energy Saving of Cold Regions Architecture of Ministry of Education, Jilin Jianzhu University, Changchun 130118, China; chengzf838@163.com (Z.C.); chiyaodan@jlju.edu.cn (Y.C.); wanghuan@jlju.edu.cn (H.W.); stone2009@126.com (X.C.); zhaoy261@163.com (Y.Z.); xiancaitang@sina.com (B.W.); wang2733567565@126.com (R.W.); zhang2505723372@163.com (Z.Z.); wangchao@jlju.edu.cn (C.W.); yangjia@jlju.edu.cn (J.Y.)

* Correspondence: lvsa@jlju.edu.cn (S.L.); hanyxt@163.com (X.Y.); Tel.: +86-0431-8456-6181 (S.L.)

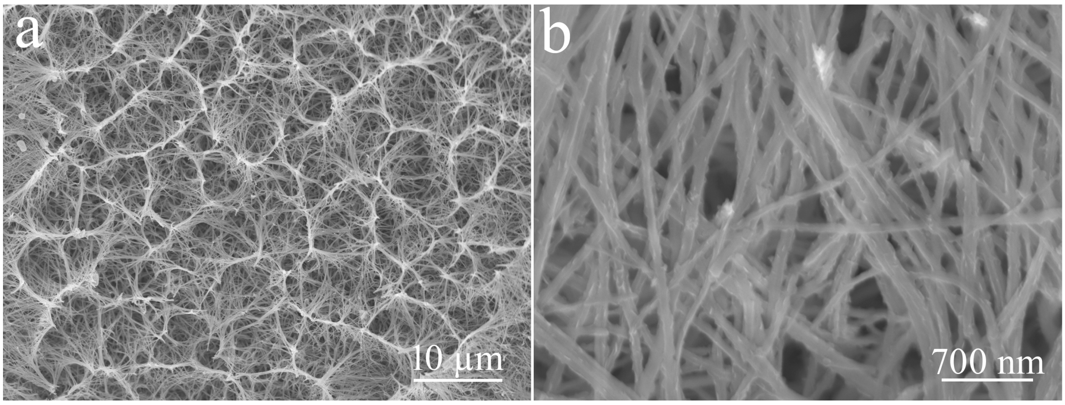

Figure S1. Low (**a**) and high (**b**) magnifification FE-SEM images of NCS electrodeposited on CuO for 1 min (S-1).

The *C*s value of CuO/NCS electrode is calculated according to the equation:

$$C_S = \frac{I \times \Delta t}{S\Delta V}$$

where C_S (F cm^{-2}) is the specific capacitance, *I* (A) is the charge and discharge current, Δt (s) is the discharging time, *S* (cm^2) is the effective area of the electrode and ΔV (V) represents the potential drop during discharge.

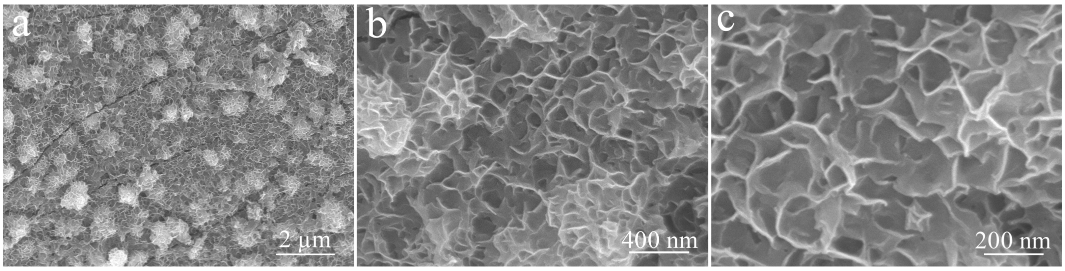

Figure S2. Low (**a**) and high (**b**,**c**) magnifification FE-SEM images of individual NCS deposited on CF at different magnifications.

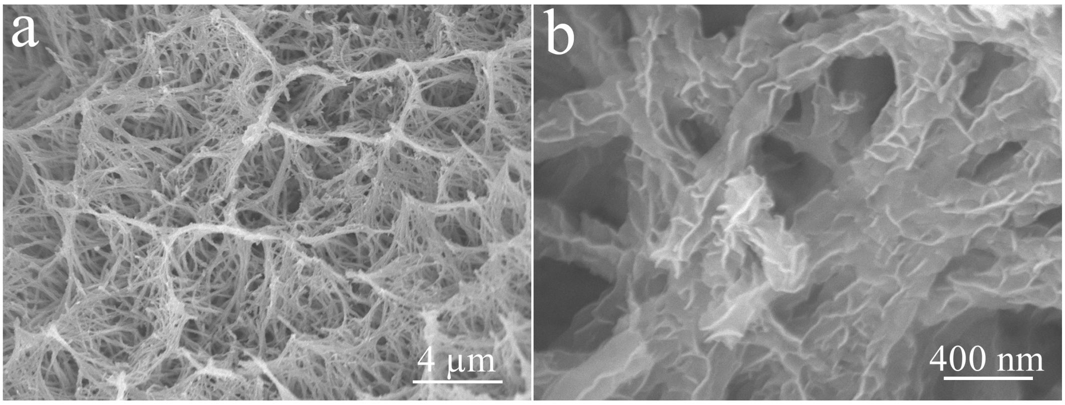

Figure S3. Low (**a**) and high (**b**) magnifification FE-SEM images of CuO/NCS at different magnifications after electrochemical performance testing.
